# Investigating the causal effect of socioeconomic status on quality of care under a universal health insurance system - a marginal structural model approach

**DOI:** 10.1186/s12913-019-4793-7

**Published:** 2019-12-23

**Authors:** Hoyune E. Cho, Lu Wang, Jung-Sheng Chen, Mochuan Liu, Chang-Fu Kuo, Kevin C. Chung

**Affiliations:** 1 Section of Plastic Surgery, Department of Surgery, University of Michigan Medical School, Ann Arbor, MI USA; 2 Department of Biostatistics, University of Michigan, Ann Arbor, MI USA; 3 Department of Rheumatology, Allergy, and Immunology, Chang Gung Memorial Hospital, 5 Fu-Hsing Street, Kwei-Shan, Taoyuan, 333 Taiwan; 4 Department of Biostatistics, University of North Carolina, Chapel Hill, NC USA; 5 Department of Rheumatology, Orthopaedics, and Dermatology, School of Medicine, University of Nottingham, Nottingham, UK; 6 Section of Plastic Surgery, Department of Surgery, Michigan Medicine, Ann Arbor, MI, 1500 East Medical Center Drive, TC 2130, Ann Arbor, MI 48109 USA

**Keywords:** Social determinants of health, Preventable hospitalization, Quality of care in the community, Universal health insurance system, Marginal structural model, Causal effect relationship

## Abstract

**Background:**

Social disparities in healthcare persist in the US despite the expansion of Medicaid under the Affordable Care Act. We investigated the causal impact of socioeconomic status on the quality of care in a setting with minimal confounding bias from race, insurance type, and access to care.

**Methods:**

We designed a retrospective population-based study with a random 25% sample of adult Taiwan population enrolled in Taiwan’s National Health Insurance system from 2000 to 2016. Patient’s income levels were categorized into low-income group (<25th percentile) and high-income group (≥25th percentile). We used marginal structural modeling analysis to calculate the odds of hospital admissions for 11 ambulatory care sensitive conditions identified by the Agency for Healthcare Research and Quality and the odds of having an Elixhauser comorbidity index greater than zero for low-income patients.

**Results:**

Among 2,844,334 patients, those in lower-income group had 1.28 greater odds (95% CI 1.24–1.33) of experiencing preventable hospitalizations, and 1.04 greater odds (95% CI 1.03–1.05) of having a comorbid condition in comparison to high-income group.

**Conclusions:**

Income was shown to be a causal factor in a patient’s health and a determinant of the quality of care received even with equitable access to care under a universal health insurance system. Policies focusing on addressing income as an important upstream causal determinant of health to provide support to patients in lower socioeconomic status will be effective in improving health outcomes for this vulnerable social stratum.

## Background

The two most important agendas to improve United States (US) healthcare are to enhance access to care and increase the quality of care delivered in an equitable manner [1–4]. Although the United States healthcare expenditures far exceeds other developed countries, the US ranks 30th in terms of morbidity and mortality [5]. Despite efforts to expand delivery of health care through the Affordable Care Act [6], 12% of the population remained uninsured in 2016 with difficult access to primary care [7]. Furthermore, middle and lower socioeconomic classes are less likely to have a regular source of care, less likely to receive preventive services, and more likely to experience delays in their care [8–11]. Studies have also found evidence for widening racial gap in health [12]. Social inequalities in access to healthcare persist in the US healthcare delivery system even with the expansion of Medicaid [8, 13, 14].

Medical care alone cannot adequately improve health or address health disparities by socioeconomic status (SES) in the US [15], and thus it is imperative to clearly delineate the causal relationship between a patient’s SES and the quality of care received, in light of the recent government proposal for cutbacks on Medicaid [16, 17]. Many studies have investigated the influences of various social determinants of health, but few have recognized the longitudinal, compounding effect of SES on health [15, 18, 19]. Furthermore, the confounding bias between variables of SES, race, and health outcomes were not investigated in the analysis [8, 9, 12, 20–26]. In fact, SES and race are deeply intertwined in their development and longitudinal continuum through multiple familial generations in the US [12, 15]. Even the direction of causality between SES, race, and health has been in debate [12, 19]. In addition, SES is correlated with the type of insurance coverage, which further leads to disparities in access to care and health outcomes [10]. Experts state that the confounding effect among race, SES, and health in the US cannot be sufficiently eliminated by statistical means [12].

In contrast to our racially diverse nation with a multi-payer insurance system, Taiwan’s population sample is homogeneous for race and insurance type, owing to the establishment of a universal, single-payer health insurance system in 1995 [27]. Covering over 99% of the residents, the National Health Insurance (NHI) provides easily accessible, equitable care [27, 28]. Utilizing the NHI Database, we designed a population-based study with marginal structural modeling to more accurately investigate the longitudinal causal influence of SES on the quality of care in the community. Regarding income as a measure of SES, we hypothesized that without the confounding bias from race and insurance type, income is not a causal factor in determining the quality of care received. Results from this study will direct health policy towards improving quality of care by providing appropriate support for patients in lower SES.

## Methods

### Data source

**Fig. 1:**
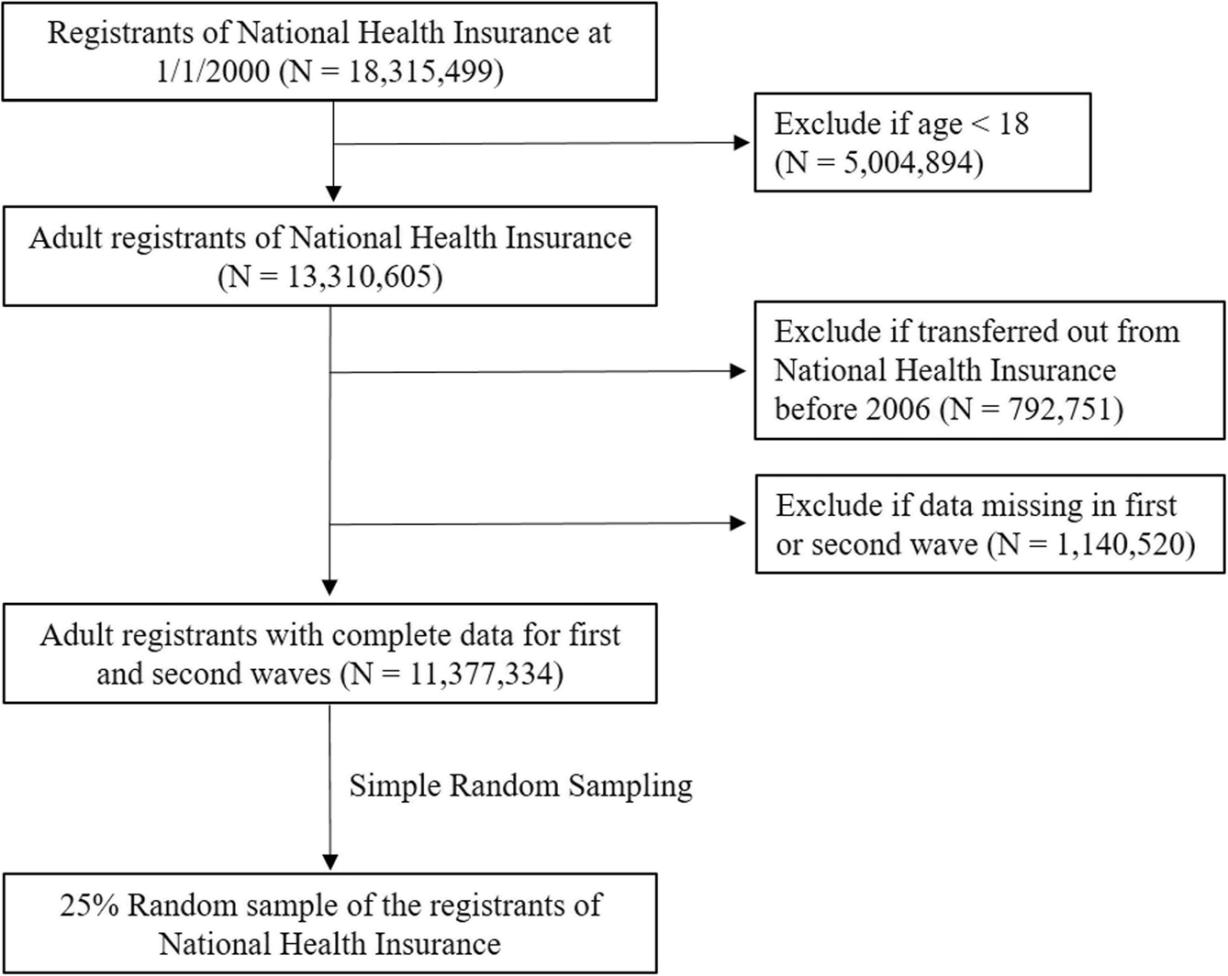
Selection flow for 25% random sample from Taiwan population

As one of the largest and most comprehensive national-level population databases in the world [29], the NHI Database contains healthcare records of 30 million residents of Taiwan, including inpatient, outpatient, and pharmacy services [30]. The NHI Database is ideal for longitudinal epidemiological investigation because each beneficiary of the NHI has a unique identification number consistent across all datasets, and can be followed through multiple clinical encounters [31]. To maximally retain the population heterogeneity to reflect the real-world impact of SES, we selected a random 25% sample from the eligible adult population in 2000 from the NHI Database by random sampling method based on Floyd’s ordered hash table algorithm to ensure an equal probability of the eligible population being selected. We also excluded patients with missing data before 2006 for appropriate censoring and to ensure two-waves worth of longitudinal data at minimum (Fig. 1). This study was approved by the Institutional Review Board at the Chang Gung Memorial Hospital.

### Overview of marginal structural modeling

Since patients’ socioeconomic status is a time-varying variable, we adopted marginal structural model (MSM) analysis to investigate the causal relationship between patients’ socioeconomic status as defined by income levels and the quality of care received measured by the rate of preventable hospitalization and the Elixhauser Comorbidity Index (ECI). MSM eliminates the confounding bias in estimating this causal relationship, where the other confounding effects vary over time from wave to wave, and these confounders may act as intermediate variables resulting from previous socioeconomic statuses at the same time [32, 33]. The observational data lack counterfactual outcomes that would have occurred under the opposite exposure level or treatment decision [34]. Thus, cross-sectional observational studies use stratification or multivariable regression analyses to address potential confounding and make causal inferences. However, with longitudinal data for which the time-varying confounders (variables that are associated with both treatment and the subsequent outcome) and treatment by indication (variables affected by prior exposure and affect future exposure levels) co-exist, these traditional analyses cannot account for the dynamic effects of the time-varying covariates appropriately to make a causal inference [35]. MSM provides an approach to balance out the potential confounding effects in a longitudinal study by creating a pseudo-population to mimic the data from a sequentially-randomized trial, and use that to estimate the average effect of a treatment or an exposure on potential outcomes [36]. We chose to use MSM to answer our research question because of these advantages that permit unbiased assessment of the causal effects of SES on the quality of care received in a longitudinal study. We conducted two separate models, each with a different outcome variable: one with the rate of preventable hospitalization and the other with the ECI.

### Variables of interest

We considered preventable hospitalization and comorbidity as representative outcome measures of the quality of care in the community [9]. A patient was considered to have had a preventable hospitalization if the primary diagnosis code associated with the inpatient stay was included in the set of diagnosis and procedure codes defined by the Agency for Healthcare Research and Quality for 11 of ambulatory care sensitive conditions [37] for which hospitalizations can be avoided with good outpatient care [20, 21, 38, 39]. We calculated each patient’s Elixhauser comorbidity index (ECI) [40] by defining it as having ≥2 ambulatory visits or one hospital admission with a corresponding diagnosis code to ensure the validity of indices greater than zero. The first model with preventable hospitalization as outcome included ECI as a covariate, and was calculated as a categorical variable with 3 levels (0, 1–3, and ≥ 4). In the second model with ECI as outcome, ECI was defined as a continuous variable and calculated at years 2004, 2007, 2010, 2013, and 2016; thus ECI was excluded as a covariate from this model. Demographic and clinical covariates included in the analysis were patients’ sex, age, income level, occupation type, urbanization of area of residence, number of outpatient visits, number of hospital admissions, and the physician density of the area of patients’ residence. Occupation categories defined by the NHI program enrollment protocol [30] and income levels in Taiwan dollars were collected directly from the NHI Database and were converted to US dollars by the mean exchange rate during each corresponding calendar year [41]. We compared demographic characteristics by income groups for each time wave by analysis of variance test and chi-squared test. In our MSM analysis, values at year 2000 for each independent variable was defined as baseline values. Then, the baseline values were used to calculate the time-varying variables at each wave by taking the average value during each time interval for continuous variables and the mode for categorical variables at each wave and two years before each wave. For example, for the number of outpatient visits variable, the average value between 2001 and 2003 was considered as the number of outpatient visits in 2003. To create income groups at each wave, the average income during the time-period up to each wave-year was used. For example, the average income from 2001 to 2008 was used to define the income level at 2009.

### Inverse probability of treatment weights

MSM can control the confounding effect of time-dependent confounders without over-adjusting by applying inverse probability of treatment weights (IPTW) [42]. At each time-point of follow-up, the probability of each patient receiving the treatment/exposure (or not receiving the treatment/exposure, whichever that actually took place) is estimated based on the baseline and time-varying covariates up to that time-point [42]. Then, patients are weighted by the inverse of their predicted probabilities of receiving the observed treatment/exposure to create a pseudo-population without the covariate imbalances. Under-represented subjects, given their previous covariate values and treatment history, receive proportionally higher weights, and vice-versa for over-represented subjects. In this pseudo-population, the potential confounders are distributed evenly and thus we can estimate causal effects [35]. To reduce the variability and improve the precision of estimation, we applied the stabilized version of IPTW weights [42, 43] as follows:

$$ \mathrm{SW}(t)=\prod \limits_{k=0}^t\frac{\mathit{\Pr}\left\{A(k)|\overline{A}\left(k-1\right),L(0)\right\}}{\mathit{\Pr}\left\{A(k)|\overline{A}\left(k-1\right),\overline{L}(k)\right\}} $$

*Pr*{*} denotes the probability function, A(*k*) represents the time-varying exposure at time *k*, $\overline{A}$ (*k*-1) represents the exposure history prior to time (k − 1), $\overline{L}$ (k) are the time-dependent covariates through time *k* that are possible mediators as well as confounders, and *L*(0) represents the vector of baseline covariates. Here, the numerator contains all covariates measured at baseline, and the denominator contains both baseline and time-varying confounders [42]. In our model, weights larger than 50 were considered extreme, and the weights were truncated at 50 in our analysis. To ensure that the confounders were balanced after applying IPTW, we compared absolute standardized mean differences across different exposure groups calculated before and after the weight application.

### Censoring/attrition

To minimize selection bias from inconsistent study cohort at multiple time points, we used censoring weights to account for any loss to follow-up in the data by calculating for the probability of remaining uncensored up to each point of follow-up [44, 45]. We fit censoring models to predict the probability that a patient remained in the study for each time-interval that the patient actually remained in the study [33]. Each subject was weighted by the IPTW multiplied by the inverse probability of censoring weight [42].

### MSM analysis

**Fig. 2:**
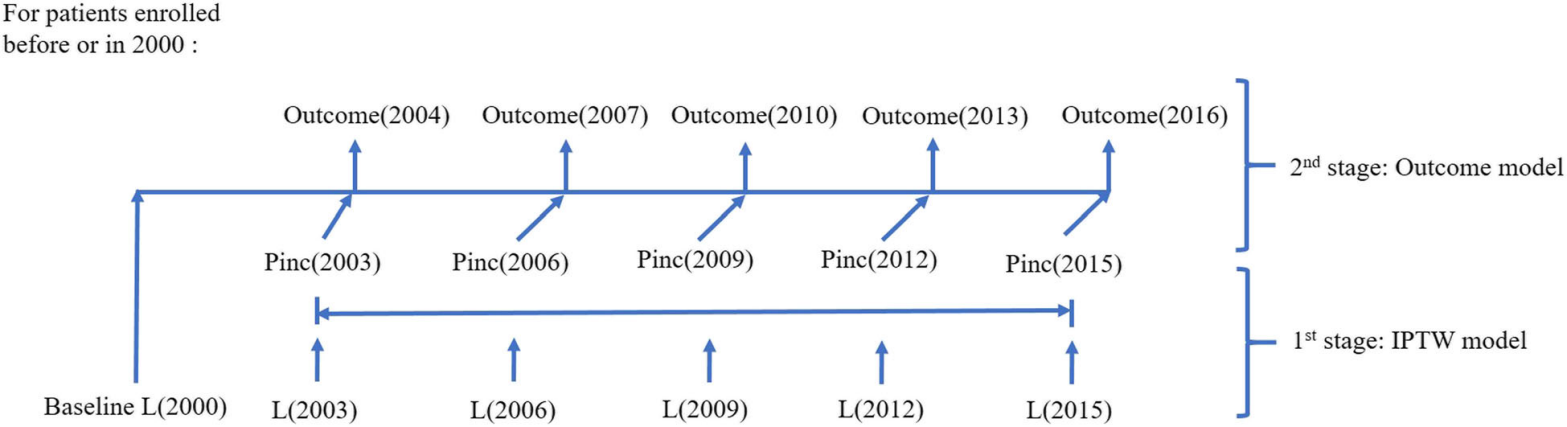
Marginal structural modeling conceptual framework. *Baseline covariates (L(0)) were used to predict the low-income group exposure at each wave. The time-varying covariates (L(t)) was used to predict the low-income exposure at each wave, Pinc(t), as the outcome variable in the first stage. Then, during the second stage, Pinc(t) was used as the independent variable to examine the causal relationship between income and preventable hospitalization and comorbidity. All models in the first stage included baseline and prior low-income status to predict Pinc(t)

We defined 5 time-waves with 3-year interval between 2000 (baseline) and 2016. The baseline values and time-varying covariates collected up to each wave-year were used to assess their relationships with the outcome variable at each wave-year (Fig. 2).

Before applying MSM, we first explored the associations of income quartiles with the outcome and found that outcomes from the higher three income quartiles were very similar. Therefore, we decided to dichotomize the income level into low-income group (<25th percentile) and high-income group (≥25th percentile).

We used a two-stage approach to estimate treatment effects from MSM (Fig. 2). During the first stage, we estimated each patient’s probability of being in his or her income group at each time point, and used the inverse of this probability as weights to balance the potential confounding owing to the observed and non-randomized income levels. To acquire the treatment weights (here low-income exposure), we fitted logistic regression models with both baseline variables and time-dependent variables up to each wave (2003, 2006, 2009, 2012, and 2015). Censoring weights were also applied by estimating the survival probability, because the most common reason for discontinuing the NHI coverage was death. Censoring was present at time = t if the patient transferred out prior to or at the next time point t + 1. The final weight for each patient was calculated by multiplying both the treatment weights and censoring weight at each time point. The numerator for treatment weighting was derived by adjusting the baseline values and previous income binary groups in the model and the denominator was derived by adjusting the baseline values and time-dependent variables. The numerator and denominator for censoring weights were obtained from the cox proportional hazard models, with the response variable in cox model as the patients’ binary censoring status.

Then, in the second stage, the causal parameter in the pseudo-population created with each individualized IPTW was recovered by fitting a weighted generalized linear model on the health outcomes (here preventable hospitalization and ECI). We applied IPTW to avoid biased estimation that happens when time-dependent confounders are inappropriately adjusted by stratification or traditional regression approaches. Furthermore, this methodology separates the time-dependent covariates confounder adjustment from the mediation adjustment in assessing the causal impact on the outcome [36, 45, 46].

## Results

### Sample characteristics - baseline

In the baseline year 2000, our study cohort included 2,844,334 patients with 32.3% in the low income group. By comparing demographic characteristics, our 25% random sample was not significantly different from the overall population (Additional file 1). Among patients in the low-income group, 59.1% lived in urban, and 34.5% in suburban areas. The distribution by place of residence for urban and suburban areas were similar between low-income and high-income groups, but the proportion of high-income group living in rural areas (10.2%) was greater than that of the low-income group (6.4%). In fact, only 16.7% (44,262/220,148) of rural dwellers were categorized as low-income. Distribution by occupation type was similar between low and high-income groups for public employees (category 1, 21.4% vs. 19.0%) and private employees (category 2, 37.6% vs. 39.3%). However, for self-employed (category 3) and those related to the military (category 4), over 99% were included in the high-income group. Approximately 93.7% of veterans and those without permanent jobs (category 6) were categorized as low-income. The composition of patient mix by ECI was similar between the two income groups. The mean number of outpatient visits (calculated per 1000 patients) was 11.3 for low-income group and 11.5 for high-income group. The mean number of hospital admissions, also calculated for every 1000 patients, was 0.11 for low-income group and 0.10 for high-income group. In other words, patients with higher income utilized ambulatory care services more frequently whereas lower-income patients required inpatient services at a higher rate (Table 1).

**Table 1:** Sample characteristics, baseline and first waves (no. (%), mean (95% CI))

	Baseline (2000)			First wave (2003)		
	Low income	High income	*P* value	Low income	High income	*P* value
	(29–586 USD)	(615–1851 USD)		(26–543 USD)	(543–2594 USD)	
N	694,162	2,150,172		711,684	2,132,650	
Place of residence						
Urban	410,251 (59.1%)	1,257,923 (58.5%)	<.001	429,044 (60.3%)	1,274,360 (59.8%)	<.001
Suburban	239,649 (34.5%)	672,101 (31.3%)		234,431 (32.9%)	632,389 (29.7%)	
Rural	44,262 (6.4%)	220,148 (10.2%)		48,209 (6.8%)	225,901 (10.6%)	
Occupation category^a^						
1	148,891 (21.4%)	408,848 (19.0%)	<.001	150,682 (21.2%)	385,223 (18.1%)	<.001
2	260,675 (37.6%)	844,689 (39.3%)		212,327 (29.8%)	853,357 (40.0%)	
3	101 (0.0%)	456,637 (21.2%)		10,839 (1.5%)	446,917 (21.0%)	
4	0 (0.0%)	420,587 (19.6%)		3201 (0.4%)	435,063 (20.4%)	
5	10,542 (1.5%)	884 (0.0%)		14,072 (2.0%)	643 (0.0%)	
6	273,953 (39.5%)	18,527 (0.9%)		320,563 (45.0%)	11,447 (0.5%)	
Elixhauser comorbidity index						
0	559,719 (80.6%)	1,755,767 (81.7%)	<.001	547,246 (76.9%)	1,675,097 (78.5%)	0.015
1–3	129,514 (18.7%)	383,916 (17.9%)		160,105 (22.5%)	450,250 (21.1%)	
≥ 4	4929 (0.7%)	10,489 (0.5%)		4333 (0.6%)	7303 (0.3%)	
Outpatient visits^b^	11.30 (11.27–11.33)	11.52 (11.50–11.53)	<.001	11.41 (11.38–11.43)	11.36 (11.35–11.38)	<.001
Inpatient Stays^b^	0.113 (0.112–0.114)	0.095 (0.094–0.095)	<.001	0.120 (0.119–0.121)	0.096 (0.096–0.097)	<.001
Physician Density of Residence^b^	1.573 (1.569–1.577)	1.712 (1.709–1.714)	<.001	1.655 (1.651–1.659)	1.809 (1.806–1.812)	<.001

^a^ Category 1 = civil servants, full-time or regularly paid personnel in governmental agencies and public schools, 2 = employees of privately owned enterprises or institutions, 3 = self-employed, other employees or paid personnel, and members of farmer and fishermen associations, 4 = military personnel, military school students, bereaved families of deceased military personnel, public service in lieu of military service, 5 = low-income citizens, 6 = veterans and dependents, and citizens without a fixed profession from other areas

^b^ Number per 1000 patients

### Sample characteristics first wave to fifth wave

The total number of patients in the study decreased from 2,844,334 in the first and second waves to 2,753,224 in the third, 2,644,668 in the fourth, and 2,538,246 in the fifth wave. Thus, the overall rate of attrition during the 16-year study period was 10.8%. The high-end limit in income ranges increased for both low and high-income groups from first wave to fourth wave, reflecting the growth of Taiwan economy over the years. The distribution of residence by urbanization stayed relatively constant throughout the study period.

The proportion of patients with ECI of 0 decreased over the years for both low and high-income groups, whereas those with index between 1 and 3 increased for both groups. There was an increasing trend in rate of health care utilization by the low-income group, as the number of outpatient visits increased from 2003 to 2015 whereas it stayed relatively constant for the high-income group. The number of inpatient admissions stayed the same for both income groups. The physician density in area of residence increased over the years for both income groups as well (Tables 1, 2, and 3).

**Table 2:** Sample characteristics, second and third waves (no. (%), mean (95% CI))

	Second wave (2006)			Third wave (2009)		
	Low income	High income	*P* value	Low income	High income	*P* value
	(28–580 USD)	(580–4077 USD)		(28–592 USD)	(592–4087 USD)	
N	711,590	2,132,744		690,034	2,063,190	
Place of residence						
Urban	435,359 (61.2%)	1,280,870 (60.1%)	<.001	429,769 (62.3%)	1,256,949 (60.9%)	<.001
Suburban	227,213 (31.9%)	630,682 (29.6%)		213,573 (31.0%)	598,487 (29.0%)	
Rural	49,018 (6.9%)	221,192 (10.4%)		46,692 (6.8%)	207,754 (10.1%)	
Occupation category^a^						
1	146,102 (20.5%)	357,037 (16.7%)	<.001	143,267 (20.8%)	351,295 (17.0%)	<.001
2	181,885 (25.6%)	887,469 (41.6%)		159,376 (23.1%)	846,449 (41.0%)	
3	19,462 (2.7%)	445,932 (20.9%)		20,309 (2.9%)	446,322 (21.6%)	
4	4097 (0.6%)	433,736 (20.3%)		3769 (0.5%)	412,798 (20.0%)	
5	17,015 (2.4%)	626 (0.0%)		18,369 (2.7%)	155 (0.0%)	
6	343,029 (48.2%)	7944 (0.4%)		344,944 (50.0%)	6171 (0.3%)	
Elixhauser comorbidity index						
0	501,825 (70.5%)	1,577,535 (74.0%)	<.001	484,908 (70.3%)	1,533,179 (74.3%)	<.001
1–3	204,453 (28.7%)	546,943 (25.6%)		201,450 (29.2%)	524,644 (25.4%)	
≥ 4	5312 (0.7%)	8266 (0.4%)		3676 (0.5%)	5367 (0.3%)	
Outpatient visits^b^	12.24 (12.21–12.27)	11.63 (11.61–11.64)	<.001	12.06 (12.03–12.09)	11.53 (11.51–11.54)	<.001
Inpatient stays^b^	0.137 (0.136–0.138)	0.104 (0.104–0.105)	<.001	0.130 (0.129–0.130)	0.101 (0.101–0.101)	<.001
Physician density of residence^b^	1.728 (1.725–1.732)	1.835 (1.832–1.837)	<.001	1.820 (1.817–1.824)	1.894 (1.891–1.896)	<.001

^a^ Category 1 = civil servants, full-time or regularly paid personnel in governmental agencies and public schools, 2 = employees of privately owned enterprises or institutions, 3 = self-employed, other employees or paid personnel, and members of farmer and fishermen associations, 4 = military personnel, military school students, bereaved families of deceased military personnel, public service in lieu of military service, 5 = low-income citizens, 6 = veterans and dependents, and citizens without a fixed profession from other areas

^b^ Number per 1000 patients

**Table 3:** Sample characteristics, fourth and fifth waves (no. (%), mean (95% CI))

	Fourth Wave (2012)			Fifth Wave (2015)		
	Low income	High income	*P* value	Low income	High income	*P* value
	(32–650 USD)	(650–5966 USD)		(37–694 USD)	(694–6125 USD)	
N	661,473	1,983,195		633,181	1,905,065	
Place of residence						
Urban	407,621 (61.6%)	1,192,881 (60.1%)	<.001	388,749 (61.4%)	1,141,552 (59.9%)	<.001
Suburban	209,417 (31.7%)	592,433 (29.9%)		201,148 (31.8%)	576,237 (30.2%)	
Rural	44,435 (6.7%)	197,881 (10.0%)		43,284 (6.8%)	187,276 (9.8%)	
Occupation category^a^						
1	141,949 (21.5%)	344,832 (17.4%)	<.001	146,710 (23.2%)	394,423 (20.7%)	<.001
2	154,881 (23.4%)	803,378 (40.5%)		135,848 (21.5%)	763,839 (40.1%)	
3	15,196 (2.3%)	445,780 (22.5%)		10,742 (1.7%)	389,919 (20.5%)	
4	3676 (0.6%)	384,836 (19.4%)		3134 (0.5%)	354,372 (18.6%)	
5	23,255 (3.5%)	22 (0.0%)		25,266 (4.0%)	8 (0.0%)	
6	322,516 (48.8%)	4347 (0.2%)		311,481 (49.2%)	2504 (0.1%)	
Elixhauser comorbidity index						
0	436,629 (66.0%)	1,405,986 (70.9%)	<.001	406,967 (64.3%)	1,333,529 (70.0%)	<.001
1–3	221,470 (33.5%)	572,353 (28.9%)		223,665 (35.3%)	567,905 (29.8%)	
≥ 4	3374 (0.5%)	4856 (0.2%)		2549 (0.4%)	3631 (0.2%)	
Outpatient visits^b^	12.30 (12.27–12.33)	11.59 (11.57–11.60)	<.001	12.43 (12.41–12.46)	11.61 (11.60–11.62)	<.001
Inpatient stays^b^	0.126 (0.125–0.127)	0.099 (0.098–0.099)	<.001	0.121 (0.121–0.122)	0.097 (0.096–0.097)	<.001
Physician density of residence^b^	1.901 (1.897–1.904)	1.955 (1.952–1.957)	<.001	1.976 (1.972–1.980)	2.018 (2.015–2.020)	<.001

^a^ Category 1 = civil servants, full-time or regularly paid personnel in governmental agencies and public schools, 2 = employees of privately owned enterprises or institutions, 3 = self-employed, other employees or paid personnel, and members of farmer and fishermen associations, 4 = military personnel, military school students, bereaved families of deceased military personnel, public service in lieu of military service, 5 = low-income citizens, 6 = veterans and dependents, and citizens without a fixed profession from other areas

^b^ Number per 1000 patients

### Analysis of causal inference by MSM

The covariates between the low-income and high-income groups were not balanced across the waves (Tables 1, 2, and 3). We compared the absolute standardized mean differences before and after applying the IPTW and confirmed that the weighted data used in MSM analysis had the covariates balanced (Additional file 2). Through MSM analysis, we found that patients in low-income group had 1.28 times greater odds of incurring a preventable hospitalization in comparison to patients in the high-income group (*p* < 0.001). In other words, patients with lower income were 28% more likely to require inpatient-level treatment of their ambulatory care sensitive condition(s). We also found that patients in low-income group had 1.04 times greater odds of having a comorbidity (*p* < 0.001) (Table 4).

**Table 4:** Analysis of causal impact of low income on health with marginal structural modeling

	Low income (<25th percentile) vs. High income (≥25th percentile)	
	Odds ratio (95% CI)	*P* value
Preventable hospitalization	1.28 (1.24–1.33)	< 0.001
Elixhauser comorbidity index	1.04 (1.03–1.05)	< 0.001

## Discussion

In our study, patients in lower income group were 28% more likely to experience a preventable hospitalization, which indicates that they have a much higher risk of acquiring diseases severe enough to require inpatient-level treatment that would have been prevented with good ambulatory care. Our finding of lower income patients having 4% higher odds for comorbidity echoes this interpretation. Our analysis demonstrated that income is a causal factor in a patient’s health status even under a universal health insurance system that grants equitable access to care. Furthermore, as the Taiwanese population is relatively homogenous in race, this finding is in a setting where racial disparities in healthcare that exist in the US is eliminated. In addition, our use of MSM shows that the relationship between a patient’s SES and quality of care received is actually causal, rather than correlational.

Past studies that have investigated the influence of various demographic and health factors on preventable hospitalization are based on observational data [8, 10, 11, 20–22, 25] and there is a concern with effect estimates that they may be biased from unobserved confounding among the variables used [46]. Moreover, these studies used cross-sectional designs that assume the magnitude of influence of each study variable is constant and takes immediate effect [46]. Rather, many of these patient and environment factors influence health at each life stage, with accumulating social advantages and disadvantages that translate to health advantages and disadvantages over time through complex causal pathways [15, 21, 46]. In fact, a systematic review comparing estimates from conventional analysis and MSM found that there is a statistically significant difference in results between the two analytic designs [35]. Thus, our result by MSM analysis can be considered to be a more accurate estimate of the true effect of a patient’s socioeconomic status on quality of care received.

Our study has established the causal impact of income on health, and that it is a social factor at the very root of health inequalities stemming from issues both inside and outside of the healthcare division [9, 23, 47]. Upstream social determinants of health have been correlated to the onset and progression of various diseases as well as to overall mortality [48], and experts stress that many of these factors influence each other as well [21]. It is well accepted that strategies to solve health disparities should address the very generators of the inequalities [23], yet in our current healthcare system, patients’ health-related social needs are seldom evaluated or addressed [48, 49]. Information on these factors along with patients’ clinical data would permit providers to tailor services and improve effectiveness of the care delivered [48]. Global health community considers universal health insurance coverage as a tool to improve access to care and further the population health, but our results suggest that providing universal health insurance is not enough to overcome the persistent inequalities in health by SES [50]. Policies that recognize income as an upstream causal determinant of health would be more effective in addressing the health disparities by SES and improve the quality of care for patients in lower socioeconomic group [51, 52].

Based on the nature of claims data, our study is subject to error from the inherent uncertainty in coding practice. Also, the validity and consistency of IPTW is based on the assumption that there are no other unmeasured confounders in MSM analysis [32], which is not guaranteed in real-life situations. Furthermore, MSM holds under the condition that the measured covariates at each time point are sufficient to adjust for confounding, which cannot be tested with observed data [44]. However, our study cohort is comprised from a national database with demonstrated validity of recorded diagnoses [53–55] that represents more than 99% of the population, and thus coding errors and the selection bias stemming from the study design are minimized. We excluded patients with missing data before 2006 for appropriate censoring and to ensure two-waves worth of data, and this may add to the selection bias. However, the excluded patients are small in proportion (10% of the registrants of the NHI in 2000, Fig. 1) and thus should not lay a significant impact on our results. Lastly, our study timeframe of 16 years captures a small portion of a patient’s lifetime [46]. Nevertheless, our results still successfully showcase the causal effect of income on a patient’s health and quality of care received with methodology to address time-varying confounding bias within longitudinal population-based data.

## Conclusion

Socioeconomic disparities in the quality of care delivered in the US persist despite the expansion of insurance coverage. Our study using data from a universal health insurance system found that income is a casual determinant of health even with equitable access to care. Patients in lower socioeconomic group would benefit greatly from policy interventions that recognize and address the upstream social determinants of health and the inequalities that emerge from them.

## Supplementary information

**Additional file 1.** Population and Study Sample Characteristics (No. (%), mean (SD)).

**Additional file 2.** Comparison of Absolute Standardized Mean Differences before and after applying IPTW.

## Data Availability

The data and materials used for this study are available from one of the corresponding authors (CFK) upon reasonable request. Public access to the data is closed.

## Abbreviations

ECI: Elixhauser comorbidity index
IPTW: inverse probability of treatment weights
MSM: marginal structural model
NHI: National Health Insurance
SES: socioeconomic status
US: United States
