# Supplementary material for: Investigating the causal effect of socioeconomic status on quality of care under a universal health insurance system - a marginal structural model approach
Source: BMC Health Serv Res. 2019 Dec 23;19:987. doi: 10.1186/s12913-019-4793-7 (PMC6929314; doi:10.1186/s12913-019-4793-7)
Supplement: Supplementary file 1 — Additional file 1. Population and Study Sample Characteristics (No. (%), mean (SD)). [file 12913_2019_4793_MOESM1_ESM.docx]

**Supplement 1. Population and Study Sample Characteristics (No. (%), mean (SD))**

|  | **Entire NHI Database at Baseline (2000)** | **25% Random Sample at Baseline (2000)** | **P value** |
| --- | --- | --- | --- |
| **Number of patients** | 11,377,334 | 2,844,334 | - |
| **Male** | 5,460,840 (48.0%) | 1,365,821 (48.0%) | 0.52 |
| **Age** | 43.2 (15.1) | 43.2 (15.1) | 0.81 |
| **Place of residence** | | | |
| **Urban** | 6,671,718 (58.6%) | 1,668,174 (58.6%) | 0.86 |
| **Suburban** | 3,648,721 (32.1%) | 911,750 (32.1%) |  |
| **Rural** | 1,056,895 (9.3%) | 264,410 (9.3%) |  |
| **Occupation category^a^** | | | |
| **1** | 2,235,182 (19.6%) | 557,739 (19.6%) | 0.36 |
| **2** | 4,422,380 (38.9%) | 1,105,364 (38.9%) |  |
| **3** | 1,823,144 (16.0%) | 456,738 (16.1%) |  |
| **4** | 1,682,935 (14.8%) | 420,587 (14.8%) |  |
| **5** | 46,223 (0.4%) | 11,426 (0.4%) |  |
| **6** | 1,167,470 (10.3%) | 292,480 (10.3%) |  |
| **Elixhauser index** | | | |
| **0** | 9,263,936 (81.4%) | 2,315,486 (81.4%) | 0.79 |
| **1-3** | 2,051,711 (18.0%) | 513,430 (18.1%) |  |
| **≥4** | 61,687 (0.5%) | 15,418 (0.5%) |  |
| **Outpatient visits^b^** | 11.5 (12.5) | 11.5 (12.5) | 0.99 |
| **Inpatient stays^b^** | 0.1 (0.4) | 0.1 (0.4) | 0.93 |
| **Physician density of residence^b^** | 1.7 (1.8) | 1.7 (1.8) | 0.28 |
| **Income in USD** | 752 (453.2) | 752 (453.2) | 0.38 |
| **Range** | 29-1851 | 29-1851 | - |
| **Outcome 1: Preventable hospitalization** | | | |
| **First wave (2004)** | 115,784 (1.0%) | 29,167 (1.0%) | 0.24 |
| **Second wave (2007)** | 159,425 (1.4%) | 40,073 (1.4%) | 0.33 |
| **Third wave (2010)** | 163,267 (1.5%) | 40,728 (1.5%) | 0.68 |
| **Fourth wave (2013)** | 160,139 (1.5%) | 40,162 (1.5%) | 0.58 |
| **Fifth wave (2016)** | 184,082 (1.8%) | 45,958 (1.8%) | 0.82 |
| **Outcome 2: Elixhauser index** | | | |
| **First wave (2004)** | 0.42 (0.84) | 0.42 (0.84) | 0.53 |
| **Second wave (2007)** | 0.53 (0.97) | 0.53 (0.97) | 0.89 |
| **Third wave (2010)** | 0.62 (1.04) | 0.62 (1.04) | 0.67 |
| **Fourth wave (2013)** | 0.73 (1.11) | 0.73 (1.11) | 0.77 |
| **Fifth wave (2016)** | 0.60 (1.01) | 0.60 (1.01) | 0.77 |

^a^ Category 1 = civil servants, full-time or regularly paid personnel in governmental agencies and public schools, 2 = employees of privately owned enterprises or institutions, 3 = self-employed, other employees or paid personnel, and members of farmer and fishermen associations, 4 = military personnel, military school students, bereaved families of deceased military personnel, public service in lieu of military service, 5 = low-income citizens, 6 = veterans and dependents, and citizens without a fixed profession from other areas.

**^b^** Number per 1000 patients.
